# Social Deprivation and Consumer-Reported Unmet Service Needs in Home- and Community-Based Services

**DOI:** 10.1093/geroni/igaf122.1134

**Published:** 2025-12-31

**Authors:** Tetyana Shippee, Romil Parikh, Nicholas Musinguzi, Benjamin Langworthy, Jack Wolf, Stephanie Giordano, Eric Jutkowitz

**Affiliations:** University of Minnesota, Minneapolis, Minnesota, United States; University of Minnesota School of Public Health, Minneapolis, Minnesota, United States; University of Minnesota, Minneapolis, Minnesota, United States; University of Minnesota School of Public Health, Minneapolis, Minnesota, United States; University of Minnesota School of Public Health, Minneapolis, Minnesota, United States; Human Services Research Institute, Cambridge, Massachusetts, United States; University of Minnesota, Minneapolis, Minnesota, United States

## Abstract

Greater social needs, such as limited transportation, are linked to poorer outcomes in Medicaid home- and community-based services (HCBS). However, their relationship with consumer-reported unmet HCBS needs–a key measure of quality– remains unclear. Understanding this relationship is critical for identifying barriers to service access and informing policy interventions. To address this gap, we examined the association between the Social Deprivation Index (SDI; range: 1-100) and unmet HCBS needs for personal care and homemaker services, considering both individual- and area-level factors. The SDI is a composite measure of seven area-level socioeconomic indicators from the U.S. Census Bureau, including poverty, rental housing, overcrowding, single-parent households, car access, education level, and unemployment. Our study included 6,558 community-dwelling adults aged 65 and older from the 2022-2023 National Core Indicators–Aging & Disability Adult Consumer Survey. Using logistic regression, we adjusted for individual-level sociodemographic and health factors. In all regression models, SDI score was not meaningfully associated with unmet needs for HCBS for personal care and homemaker chores (p > 0.05). However, individuals with better self-rated health had significantly lower odds of reporting unmet needs (odds ratios: 0.19-0.61; p < 0.001). Similarly, those living with family or friends (versus alone) had lower odds of unmet needs (odds ratios: 0.40-0.70; p < 0.05). These findings highlight the importance of person-level factors in HCBS access rather than area-level deprivation. Future research should investigate whether place-based factors predict the geographic distribution of unmet HCBS needs and other HCBS outcomes at the zip code level.

